# Coordination Programming of Photofunctional Molecules

**DOI:** 10.3390/molecules18044091

**Published:** 2013-04-05

**Authors:** Ryota Sakamoto, Shinpei Kusaka, Mikihiro Hayashi, Michihiro Nishikawa, Hiroshi Nishihara

**Affiliations:** Department of Chemistry, Graduate School of Science, The University of Tokyo, 7-3-1, Hongo, Bunkyo-ku, Tokyo 113-0033, Japan

**Keywords:** coordination programming, photochemistry, transition metal complexes, organic electronics, photoisomerization, fluorescence

## Abstract

Our recent achievements relating to photofunctional molecules are addressed. Section 1 discloses a new concept of photoisomerization. Pyridylpyrimidine-copper complexes undergo a ring inversion that can be modulated by the redox state of the copper center. In combination with an intermolecular photoelectron transfer (PET) initiated by the metal-to-ligand charge transfer (MLCT) transition of the Cu(I) state, we realize photonic regulation of the ring inversion. Section 2 reports on the first examples of heteroleptic bis(dipyrrinato)zinc(II) complexes. Conventional homoleptic bis(dipyrrinato)zinc(II) complexes suffered from low fluorescence quantum yields, whereas the heteroleptic ones feature bright fluorescence even in polar solvents. Section 3 describes our new findings on Pechmann dye, which was first synthesized in 1882. New synthetic procedures for Pechmann dye using dimethyl bis(arylethynyl)fumarate as a starting material gives rise to its new structural isomer. We also demonstrate potentiality of a donor-acceptor-donor type of Pechmann dye in organic electronics.

## 1. 2-Pyridylpyrimidine-Cu Complexes: Visible Light-Induced Pyrimidine Ring Rotation and Reversible Cu^II^/Cu^I^ Electrochemical Potential Switching

### 1.1. Introduction

Redox switching in the Cu^II^/Cu^I^ complex is accompanied by a change in the coordination environment [1,2,3,4,5,6,7,8,9,10,11,12,13,14,15]. Cu^I^ strongly prefers the tetrahedral coordination, whereas Cu^II^ accepts several coordination numbers and modes, including square-planar, triagonal bipyramidal, and octahedral. The drastic change in the coordination environment is attractive for the construction of molecular systems that exhibit bistability [16,17,18,19,20,21,22,23,24,25,26,27].

**Figure 1:**
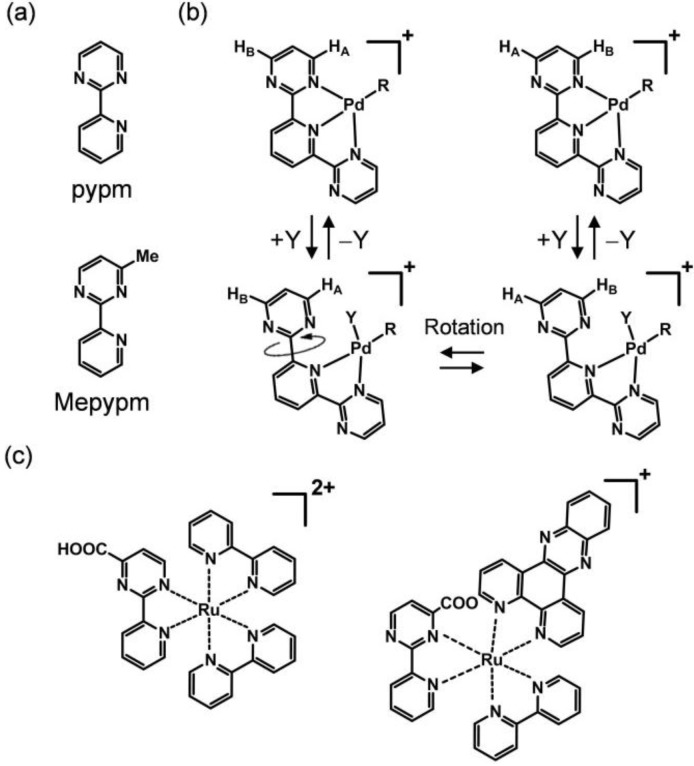
(**a**) Structure of 2-(2'-pyridyl)pyrimidine (pypm). (**b**) Ring rotation of a metal complex bearing a pypm-type ligand. (**c**) Two possible isomers of a Ru complex possessing a substituted pypm ligand. Geometric isomers are omitted for clarity.

2-(2'-Pyridyl)pyrimidine (pypm, Figure 1a) and its derivatives act as bidentate ligands with several typical characteristics. First, they possess a low-lying π* orbital. This feature leads to the expression of metal-to-ligand charge transfer (MLCT) transitions, which often emerge in the visible region. For example, a [Ru^II^(Mepypm)_3_]^2+^ cation (Mepypm = 4-methyl-2-(2'-pyridyl)pyrimidine) shows an MLCT band at *λ*_max_ = 451 nm [28]. Second, thermal rotation of the pyrimidine ring can be defined in the complex form (Figure 1b). Vrieze and coworkers demonstrated this using a Pd^II^ complex [29]. Third, the introduction of substituents on the pyrimidine ring can result in desymmetrization, giving rise to two isomeric forms upon complexation (Figure 1c). Spiccia and coworkers synthesized a pypm derivative bearing a carboxyl group at the 4 position, and considered isomers produced using its Ru^II^ complexes [30]. We note that the ring rotation is associated with the desymmetrization, and it can be recognized as a kind of linkage isomerization [31,32,33,34,35].

**Figure 2:**
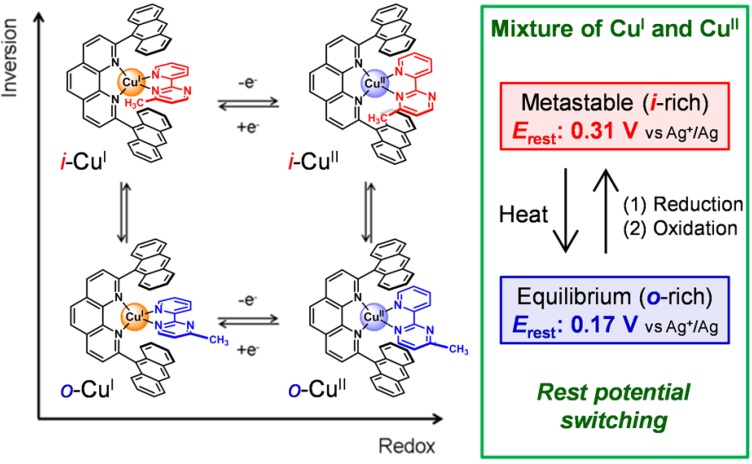
Conceptual illustration showing the rest potential switching caused by repeatable, external-stimuli-induced changes in the ratio of the four stable isomers *i*-Cu^I^, *o*-Cu^I^, *i*-Cu^II^, and *o*-Cu^II^ in a 4-methyl-2-(2′-pyridyl)pyrimidine copper complex; the chemical structures of the isomers are displayed on the left, using a square scheme.

By synergistically exploiting the peculiar characteristics of Cu complexes and pypm ligands, we have fabricated electrochemical linkage isomerization systems [36,37,38,39,40,41]. One example of this is shown in Figure 2. This heteroleptic Cu^I^ complex composed of 2,9-dianthracenylphenanthroline and Mepypm possesses two isomers; inner (*i-*), and outer (*o-*) forms, in accordance with the orientation of the methyl group. Henceforth, the *i*- and *o*-isomers of **1·BF_4_** in the Cu^I^ and Cu^II^ states are abbreviated as *i*-Cu^I^, *o*-Cu^I^, *i*-Cu^II^, and *o*-Cu^II^. The abundance ratio of the two isomers in solution was nearly 1:1 (*i*-Cu^I^:*o*-Cu^I^ = *ca.* 2:1 to 3:1) in the tetrahedral Cu^I^ state. In contrast, the *o*-form was dominant in the square-planar Cu^II^ state, because the steric repulsion between the two ligands was significant in the *i*-isomer. Therefore, reversible redox switching with respect to the Cu^II^/Cu^I^ redox couple triggered linkage isomerization. The system exhibited not only oxidation-triggered isomerization (*i*-Cu^I^− e^−^→*o*-Cu^II^) but also electrode rest potential switching; this was due to repeatable, external-stimuli-induced changes in the ratio of the four stable isomers *i*-Cu^I^, *o*-Cu^I^, *i*-Cu^II^, and *o*-Cu^II^ that occurred under a mixture of Cu^I^ and Cu^II^ complexes when the metastable state was trapped.

**Figure 3:**
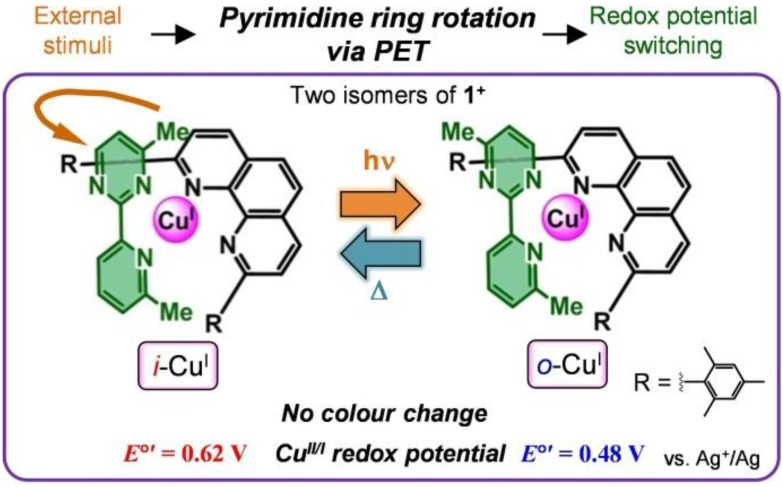
Schematic illustration of the photo-driven ring rotation of the pyrimidine moiety, and the accompanying change in the redox potential of the Cu^II^/Cu^I^ couple in **1·BF_4_**. Adapted with permission from [42]. Copyright (2012) American Chemical Society.

As described in this section in detail, our next study sought to create a ring rotation system driven by photochemical stimuli. Photochemical molecular bistability is advantageous over other bistability systems that use other stimuli, because of its practical reversibility, the fact that it can be controlled via changes in the intensity of light, and its selectivity for irradiation wavelengths. A conceptual illustration of the present study is shown in Figure 3 [42]. This system was based on photoinduced electron transfer (PET) [3,4,43]. PET is a phenomenon in which a photoexcited molecule undergoes either electron subtraction from, or electron donation to, another redox-active molecule. In fact, several electrochemical linkage isomerization molecules have shown PET-induced linkage isomerization [22,23]. Here, the molecular design, structure, photoinduced ring rotation, and additional functionality (conversion of light stimuli into electrochemical potential) are discussed.

### 1.2. Ligand Design, Structure, and Electrochemical Ring Rotation

**Figure 4:**
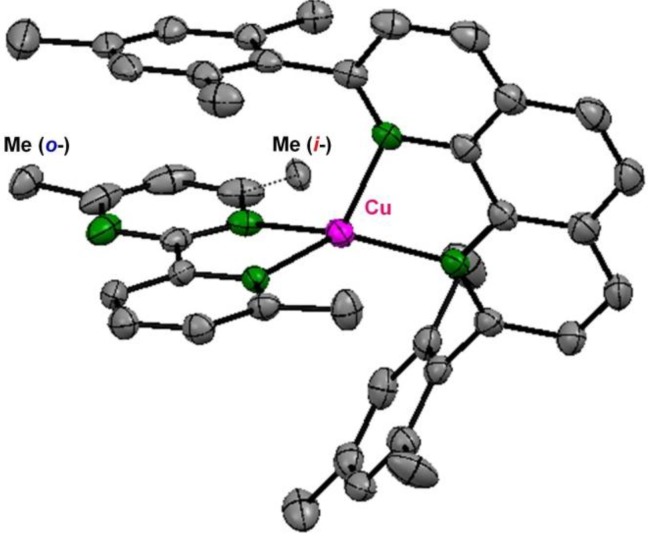
ORTEP drawing of **1·BF_4_·CH_2_Cl_2_·0.5hexane** with thermal ellipsoid sets at the 50% probability level. Hydrogen atoms, counter anions, crystal solvents, and one of the two crystallographically independent complex cations are omitted for clarity. Adapted with permission from [42]. Copyright (2012) American Chemical Society.

The structure of the Cu complex was carefully designed to enhance the optical sensitivity. A long photoexcitation lifetime is preferable for PET. The Cu^I^-diimine-type complexes feature relatively long-lived MLCT photoexcited states, and the lifetime depends significantly on the coordination structure; the substituents on the *α* position are reported to provide longer MLCT lifetimes by inhibiting the structural relaxation, and/or preventing additional solvent coordination [3,4,44,45,46,47,48,49,50,51,52]. Taking this into account, in this study we employed 4-methyl-2-(6′-methyl-2′-pyridyl)pyrimidine as a pypm-type ligand. In addition, 2,9-dimesityl-1,10-phenanthroline was adopted as an auxiliary ligand, to form a heteroleptic complex [53,54,55]. The bulky mesityl group was also expected to yield an extended MLCT lifetime. Using these two ligands, we synthesized a new heteroleptic Cu^I^-pypm complex, **1·BF_4_** (Figure 3). X-ray structural analysis of **1·BF_4_·CH_2_Cl_2_·0.5·hexane** revealed that both *i*- and *o*-isomers coexisted (Figure 4). Occupancy refinement with respect to the disordered part yielded an abundance ratio of *i*-Cu^I^:*o*-Cu^I^ = 30:70. This ratio was consistent with that measured in solution (using ^1^H-NMR spectroscopy) in the temperature range of 200–300 K.

### 1.3. Electrochemistry

**Figure 5:**
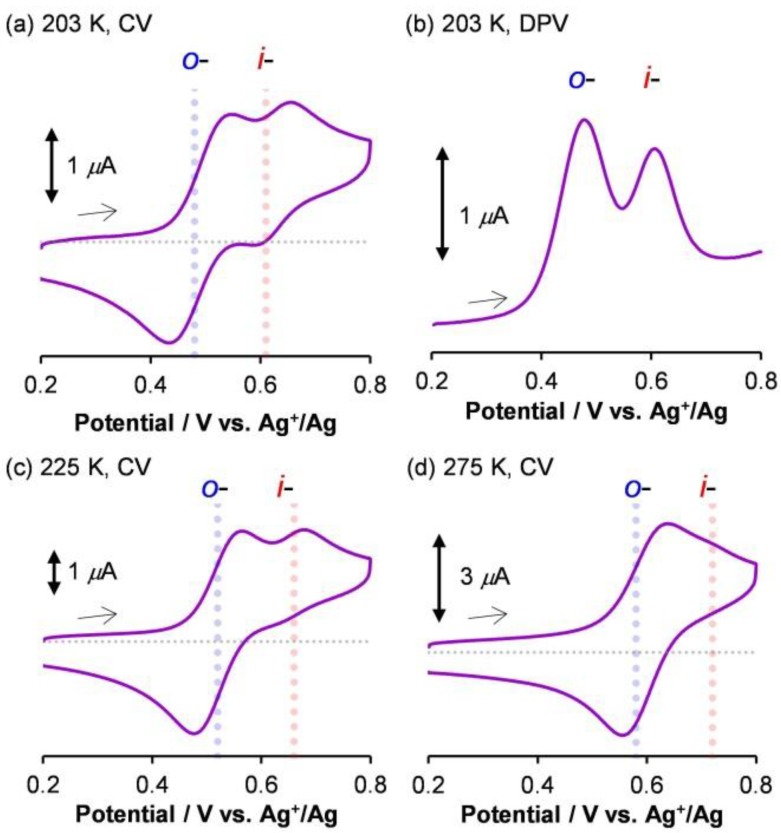
Electrochemical measurements of **1·BF_4_** (0.45 mM) in 0.1 M Bu_4_NBF_4_−CH_2_Cl_2_. (**a**,**c**,**d**) Cyclic voltammograms measured using a scan rate of 50 mV s^–1^ at 203 K (**a**), 225 K (**c**), and 275 K (**d**). (**b**) Differential pulse voltammogram measured under the same conditions as those used in (**a**). Adapted with permission from [42]. Copyright (2012) American Chemical Society.

To gain knowledge about the redox-mediated pyrimidine rotation of **1·BF_4_**, variable temperature cyclic voltammetry was conducted. Cyclic voltammetry was performed on a BAS ALS750A analyzer. Glassy carbon was used as a working electrode, platinum wire was utilized as a counter electrode, and the Ag/AgClO_4_ redox couple was exploited in a reference electrode (0.01 M AgClO_4_ in 0.1 M Bu_4_NClO_4_/acetonitrile). A standard one-compartment electrochemical cell was equipped with a USP-203-A UNISOKU cryostat so that precise temperature control was attained. All measurement was conducted under an Ar atmosphere. Figure 5a shows a cyclic voltammogram of **1·BF_4_** (0.45 mM) in 0.1 M Bu_4_NBF_4_−CH_2_Cl_2_ at 203 K, which revealed two reversible waves at *E*^0'^ = 0.48 V and 0.62 V *vs.* Ag^+^/Ag. The waves were assigned to the Cu^II^/Cu^I^ couples of the *o*- and *i*-isomers, respectively, because a bulky substituent around the Cu center destabilized the Cu^II^ square-planar geometry, thereby shifting the redox potential in the positive direction [56,57,58,59]. A differential pulse voltammogram yielded an abundance ratio (*i*-Cu^I^:*o*-Cu^I^ = 30:70) consistent with that determined using ^1^H-NMR spectroscopy (Figure 5b). At a higher temperature of 225 K, the cathodic current of the *i-*isomer significantly decreased (Figure 5c), indicating that the thermodynamic stability of *o*-Cu^II^ was much higher than that of *i*-Cu^II^. This was due to the steric repulsion, and the fact that the timescale of the transition from *o*- to *i*- was comparable with the cyclic voltammetry sweep rate [56,57,58,59]. The appearance of the voltammogram at 275 K indicated the existence of a single reversible redox process, suggesting that the interconversions were sufficiently rapid in both the copper(II) and copper(I) states (Figure 5d).

**Figure 6:**
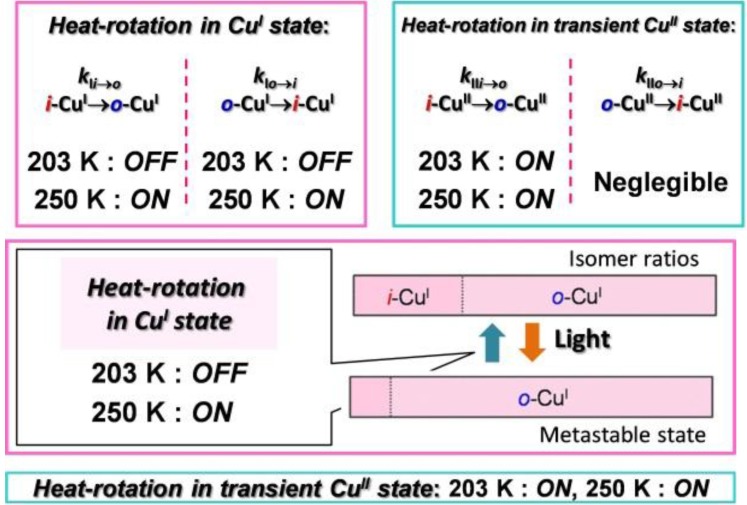
Conceptual diagram summarizing the rotation kinetics described in this section.

The first-order rate constants for the *i*-→*o*- and *o*-→*i*- rotations in the Cu^I^ state (*k*_I*i→o*_ and *k*_I*o→i*_) and Cu^II^ state (*k*_II*i→o*_ and *k*_II*o→i*_) were quantified via simulation of the cyclic voltammograms at various temperatures and scan rates using the Digisim 3.03a software (BAS Inc.). *k*_I*i→o*_ was < 10^–4^ s^–1^ at 203 K, which indicated that the rotational motion was decelerated substantially. This set of conditions was defined as the “rotation-*OFF* state” (Figure 6). At 250 K, *k*_I*i→o*_ increased to give values of the order of 10^–1^ s^–1^, indicating that the rotation was sufficiently activated (producing the “rotation-*ON* state”). Similarly, the first-order rate constant for the *o*-→*i*- rotation *k*_I*o*__→*i*_ was < 10^–4^ s^–1^ at 203 K (rotation-*OFF* state), while heating to 250 K accelerated *k*_I*o*__→*i*_ to give values of the order of 10^–1^ s^–1^ (rotation-*ON* state). In contrast, *k*_II*i→o*_ was higher than 10^–1^ s^–1^ even at 203 K (rotation-*ON* state), which indicated that the *i*-→*o*- rotation pathway in the Cu^II^ state was still active at low temperatures. *k*_II*o*→*I*_ was negligible (*k*_II*o*→*I*_ ~ 0) over the entire 200–300 K range.

### 1.4. Photophysical Properties

**Figure 7:**
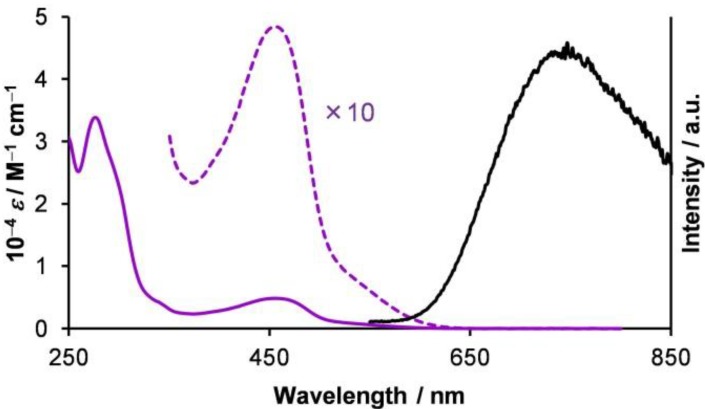
UV-vis spectra of **1·BF_4_** in CH_2_Cl_2_ at room temperature: absorption spectrum in the dark (purple); absorption spectrum in the dark, ×10 (dashed purple); emission spectrum (black). Adapted with permission from [42]. Copyright (2012) American Chemical Society.

To investigate the fueling of the rotational isomerization of **1·BF_4_** with light illumination, we examined the absorption and luminescence spectra of **1·BF_4_** (Figure 7). The characteristic absorption band in the visible region (*λ*_max_ = 456 nm, *ε* = 4.8 × 10^3^ M^–1^ cm^–1^) was attributed to the ^1^MLCT transition, which typically appears in the bis(diimine)copper(I) complex family [3]. We note that the spectrum contained contributions from both *o*-Cu^I^ and *i*-Cu^I^. Careful analysis disclosed that their absorption spectra were similar.

A broad emission band from **1·BF_4_** in dichloromethane was observed across the visible and near-IR region, with a maximum at *λ*_max_ = 750 nm (Figure 7). This emission likely arose predominantly from the *i*-isomer, because bulky substituents near the Cu^I^ coordination sphere significantly increase the lifetime of the photoexcited state, as well as the emission efficiency [3].

**Figure 8:**
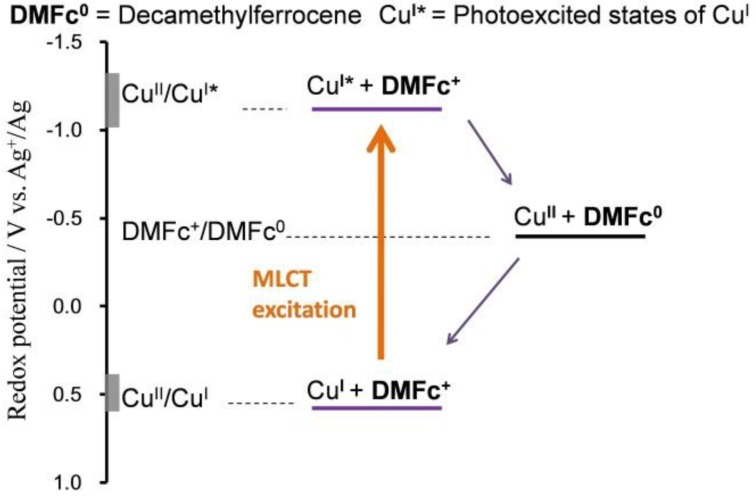
Diagram for the redox potentials of **1·BF_4_** in the ground and photoexcited states, and DMFc^+^.

A photoexcited state has a much lower reduction potential and a much higher oxidation potential than the ground state; this drives PET behavior. Figure 8 shows the reduction potentials for **1·BF_4_** in the photoexcited and ground states, which were calculated using the *E*^0'^ values of the Cu^II^/Cu^I^ couple, and the emission maximum [60]. In this scheme, a decamethylferrocenium cation (DMFc^+^, *E*^0'^ = −0.41 V *vs.* Ag^+^/Ag) can serve an oxidizing reagent suitable for our system; DMFc^+^ can undergo PET with photoexcited **1·BF_4_**, giving rise to DMFc, and the Cu^II^ state (Figure 8). In contrast, DMFc^+^ has nothing to do with **1·BF_4_** in the ground state.

### 1.5. Photodriven Rotation of the Pyrimidine Ring

**Figure 9:**
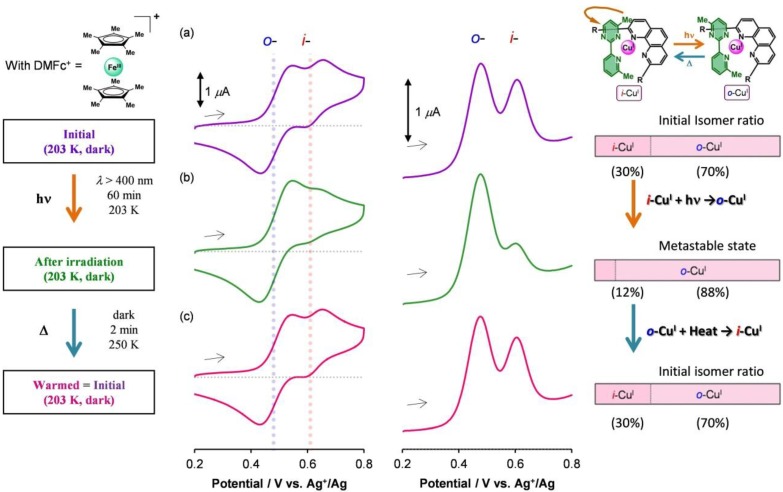
Cyclic voltammograms and differential pulse voltammograms for **1·BF_4_** (0.45 mM) in 0.1 M Bu_4_NBF_4_−dichloromethane containing 1.8 mM DMFcBF_4_ (**a**) at 203 K in the dark, (**b**) after 60 min of visible-light irradiation (*λ* > 400 nm) at 203 K, and (**c**) after 2 min of heating at 250 K in the dark. The molar ratios of i-Cu^I^ and o-Cu^I^ are represented on the right as horizontal bars. Adapted with permission from [42]. Copyright (2012) American Chemical Society.

In the presence of 4 equiv. of DMFc^+^ (BF_4_^−^ salt, 1.8 mM), the cyclic voltammograms and differential pulse voltammograms for **1·BF_4_** (0.45 mM) changed shape significantly under photoirradiation. The procedure and results are summarized in Figure 9. Light illumination was performed using a MAX-302 xenon lamp (Asahi Spectra) equipped with an optical fiber with a long-pass filter (cut-on 400 nm). We note that neither the absence of DMFc^+^ nor storage in the dark in the presence of DMFc^+^ produced changes in the voltammogram. Before photoirradiation, two redox waves assignable to the Cu^II^/Cu^I^ couple of the *i*- and *o*-isomers were observed at 203 K, in the ratio of 30:70 (Figure 9a). Under photoirradiation with visible light (*λ* > 400 nm) at 203 K for 60 min, the redox wave corresponding to the *o*-isomer increased in intensity (Figure 9b). The shapes of the voltammograms observed after photoirradiation did not change after incubation at 203 K in the dark for 10 min, confirming that the photogenerated state was not a transient state. However, subsequent heating for 2 min at 250 K in the dark recovered the initial voltammograms (Figure 9c), which indicated that the thermal relaxation of the photoinduced metastable state to the ground state had occurred. The molar ratios of *i*-Cu^I^ and *o*-Cu^I^ in the initial, photoirradiated, and thermally relaxed states were 30:70, 12:88, and 30:70, respectively [56,57,58,59]. This series of changes in the voltammograms was found to be repeatable.

### 1.5. Mechanism for Photodriven Rotation of the Pyrimidine Ring

**Figure 10:**
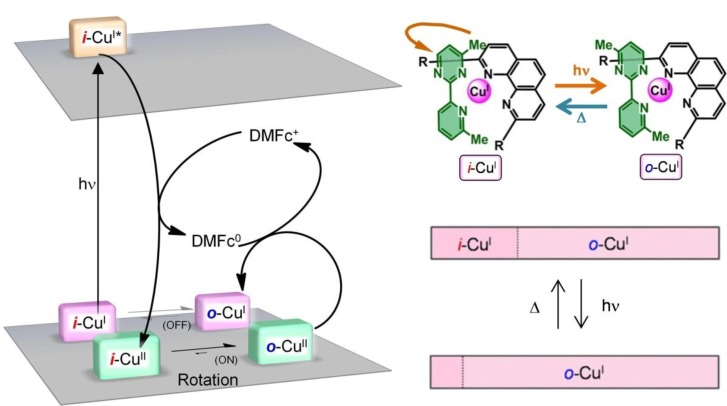
Schematic representation of the PET-driven *i*-Cu^I^-to-*o*-Cu^I^ ligand geometry isomerization of **1·BF_4_** in the presence of DMFc^+^. The reversible changes in the molar ratios of the isomers upon light irradiation and heating are illustrated in the bottom panels. Adapted with permission from [42]. Copyright (2012) American Chemical Society.

Figure 10 shows a schematic illustration of the photoinduced ring rotation system. The *i*- and *o*-isomers coexisted in the initial Cu^I^ state (*i*-Cu^I^:*o*-Cu^I^ = 30:70). The photoirradiation induced PET from the excited *i*-Cu^I^ state (*i-Cu^I^) to the electron acceptor (DMFc^+^). The oxidation potential of *i-Cu^I^ was −1.0 V, whereas the reduction potential of DMFc^+^ was −0.4 V. The *i*-Cu^II^ generated in the course of the PET isomerized to *o*-Cu^II^ for two reasons: (i) the pyrimidine ring rotation in the Cu^II^ state was not frozen at 203 K (*k*_II*i*→*o*_ ~ 10^–1^ s^–1^, rotation-*ON* state), and (ii) *i*-Cu^II^ was thermodynamically unfavorable, because of the steric repulsion. Finally, *o*-Cu^II^ was reduced to *o*-Cu^I^ via a back electron transfer reaction from DMFc. The net process resulted in the conversion of *i*-Cu^I^ to *o*-Cu^I^ upon photoirradiation. The photorotation induced a change in the abundance ratio of the *i*- and *o*- isomers (*i*-Cu^I^:*o*-Cu^I^ = 12:88). The molar ratio deviated from the thermodynamic equilibrium for the Cu^I^ state, but this state was trapped kinetically (*k*_I*o*→*i*_ ~ 10^–4^ s^–1^). Heating to 250 K provided sufficient activation for the *o*- to *i*- rotation (*k*_I*o*→*i*_ ~ 10^–1^ s^–1^), so that the thermodynamic equilibrium was restored (*i*-Cu^I^:*o*-Cu^I^ = 30:70). As a result, we attained reversible photochemical pyrimidine ring rotation, which was accompanied by a change in the redox potential shift (*ΔE*^0'^ = 0.14 V) (Figure 3). Generally, the photochromic molecule undergoes a significant color change upon isomerization, which involves the reconstruction of the electronic state [61]. Our present photo-driven rotation system worked without a significant color change. Therefore, the present method allows the creation of photodriven materials using another methodology.

### 1.6. Conclusions

We demonstrated the conversion of light stimuli into electrochemical potential via reversibly working artificial molecular rotation in a copper-pyrimidine complex, **1·BF_4_**. A key feature of the present system is that the population of bistable isomers, *i*-Cu and *o*-Cu, possessing different redox potentials (*ΔE*^0'^ = 0.14 V), is reversibly converted by light and heat stimuli through a PET process with DMFc^+^ as a redox mediator. Generally, photodriven bistable material changes are accompanied by significant color changes, which involve light absorption efficiency and reconstruction of the electronic state. On the other hand, our present system works without a significant color change, thereby providing a further methodology to construct photodriven materials. 

## 2. A Brightly Luminescent Heteroleptic bis(dipyrrinato)zinc(II) Complex

### 2.1. Introduction

**Figure 11:**
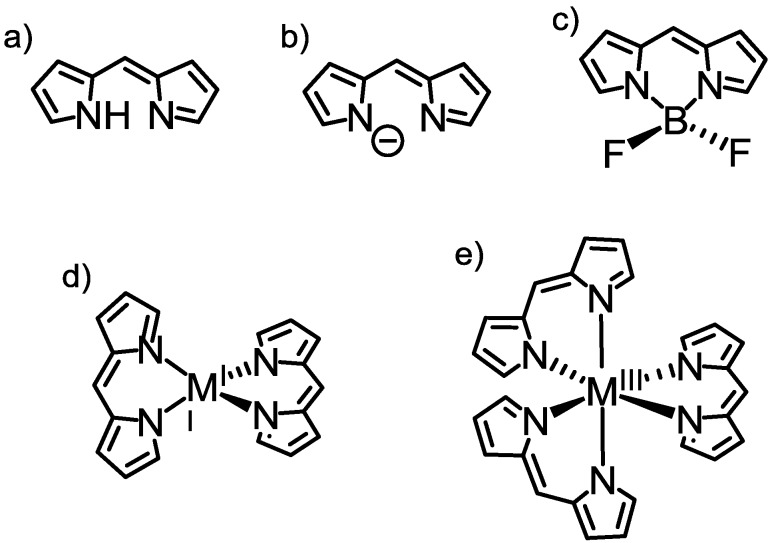
(**a**) Dipyrrin, (**b**) dipyrrinato ligand, (**c**) BODIPY, (**d**) bis(dipyrrinato)metal complex, and e) tris(dipyrrinato)metal complex.

Dipyrrin, or dipyrromethene (Figure 11a), comprises two pyrrole rings bridged by a methine carbon. Dipyrrin was originally studied as an intermediate for the synthesis of porphyrins, but chemistry was developed for dipyrrin itself after the discovery of its neutral complexes [62,63]. Dipyrrin can serve as a monoanionic bidentate ligand upon deprotonation (dipyrrinato ligand, Figure 11b). The boron difluoride complex of dipyrrin, or 4,4-difluoro-4-bora-3a,4a-diaza-*s*-indacene, known as BODIPY, is the most well known dipyrrin complex (Figure 11c). Since the first report on BODIPY in 1968 [64], it has acquired popularity as a dye molecule. Plain dipyrrin features an intense absorption in the visible region (*λ*_max_~500 nm) that is assignable to the ^1^π-π* transition; however, the intense absorption does not lead to bright fluorescence. In sharp contrast, BODIPY fluoresces brightly from the ^1^π-π* excited state; the fluorescence quantum yield often reaches unity. Due to its excellent stability against light and moisture, among other properties, BODIPY has been used in a wide range of fields, in applications including laser dyes [65,66,67], chemosensors [68,69,70,71,72], biological probes [73,74,75,76], and solar cells [77,78,79].

**Figure 12:**
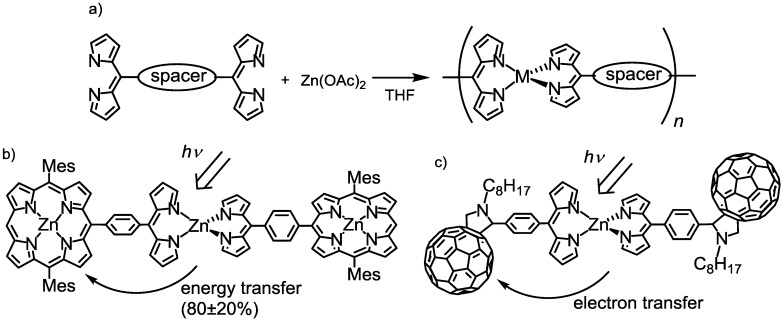
(**a**) Coordination polymer based on a dipyrrinato-metal complex. (**b**,**c**) Self-assembled donor-accepter systems that express either energy or electron transfer.

Dipyrrin can also accept various types of metal ions [63]: Fe(III), Co(III), Ni(II), Pd(II), Cu(II), Zn(II), Ga(III), and Sn(II) give rise to bis- and tris(dipyrrinato)metal complexes (Figure 11d,e). One of the most important features of dipyrrinato-metal complexes is that the coordination bonds can be generated in a self-assembled fashion, and can form supramolecular architectures. We note that these functions cannot be reproduced by BODIPY. Maeda and coworkers developed one-dimensional coordination polymers simply by mixing zinc acetate and bridging bis(dipyrrin) in tetrahydrofuran at room temperature (Figure 12a) [80], and Lindsey and coworkers reported a light-harvesting molecular array in which efficient energy transfer occurred from the central bis(dipyrrinato)zinc(II) complex to the peripheral porphyrin (Figure 12b) [81]. Guldi and coworkers reported a photoinduced charge-separation system using a bis(dipyrrinato)zinc(II) complex, and fullerene (Figure 12c) [82]. In these molecular systems, the bis(dipyrrinato)zinc(II) complex served as a glue for the construction of the triads. At the same time, it functioned as a photosensitizer. Similar to dipyrrin and BODIPY, this series of complexes possessed a ^1^π-π* band that absorbed visible light efficiently.

**Figure 13:**
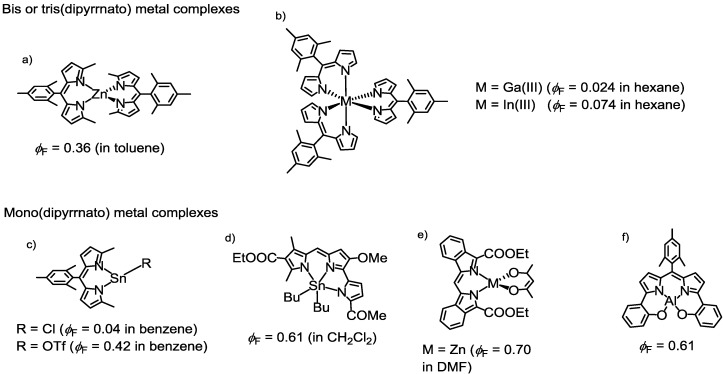
Fluorescent mono, bis, and tris(dipyrrinato) metal complexes.

In contrast with BODIPY, which has been extensively studied, basic investigations for dipyrrinato-metal complexes have not been performed, and practical applications have not been fully developed. One possible reason for this is their poorer photochemical activity, which is reflected in their low fluorescence quantum yields. Much effort has been made to improve the photochemical activity of dipyrrinato-metal complexes. Lindsey and coworkers reported a bis(dipyrrinato)zinc(II) complex bearing an bulky aryl group at the *meso*-position (Figure 13a). The bulkiness inhibited the thermal rotation of the aryl group, leading to a moderate increase in the fluorescence quantum yield (0.006→0.36) in toluene [83]. Cohen and co-workers reported weakly fluorescent tris(dipyrrinato) group 13 metal complexes (Figure 13b) [84]. Many mono(dipyrrinato)metal complexes have been reported to be highly fluorescent (Figure 13c–f) [85,86,87,88], but they are often sensitive to temperature or moisture. In addition, mono(dipyrrinato) metal complexes cannot be differentiated from BODIPY derivatives, and they have not been fully exploited for the fabrication of supramolecular assemblies. Thus, there is room for further work on improving the fluorescence quantum yield of dipyrrinato-metal complexes.

### 2.2. Strategy to Improve the Fluorescence Quantum Yield of bis(dipyrrinato)zinc(II) Complexes

**Figure 14:**
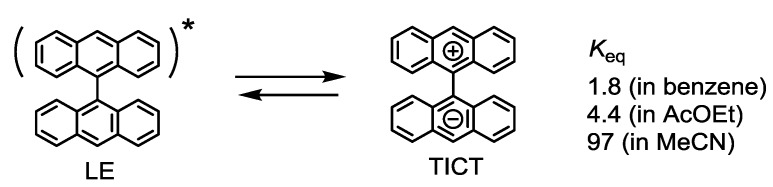
Charge separation in 9,9'-bianthryl.

Several bichromophoric systems show charge-separated (CS) states [89,90,91,92,93,94]. For example, the photoexcitation of 9,9'-bianthryl produces a locally excited state (LE) (Figure 14). The LE is in thermodynamic equilibrium with a twisted intramolecular excited state (TICT) [89,90], which is generated by an interligand one-electron transfer. Such phenomena are labeled as “photoinduced symmetry-breaking charge separation” [91,92]. The thermodynamic equilibrium between LEs and TICTs strongly depends on the solvent polarity; TICTs are dominant in polar solvents [90].

**Figure 15:**
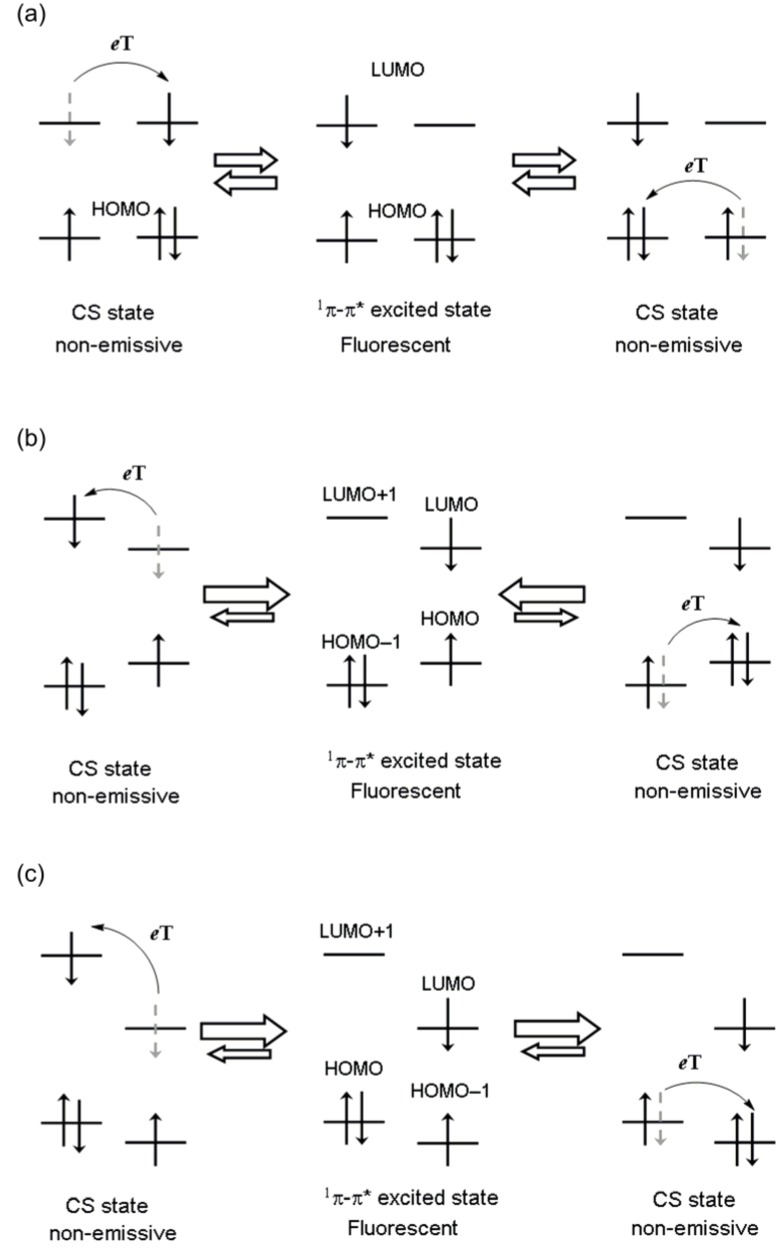
Schematic illustration of charge-separation hypothesis, for (**a**) a homoleptic complex, (**b**) a heteroleptic complex with a favorable frontier orbital order, and (**c**) a heteroleptic complex with an unfavorable frontier orbital order.

The bis(dipyrrinato)zinc(II) complex can be also regarded as a bichromophoric molecule, where two dye entities (dipyrrinato ligands) are ligated by a zinc(II) ion. Here, we propose the hypothesis that charge separation may occur between the two ligands via the ^1^π-π* excited state, and the formation of the CS states may suppress the fluorescence of the bis(dipyrrinato)zinc(II) complex. A schematic illustration of this hypothesis is shown in Figure 15a.

To destabilize the charge-separated states, we designed heteroleptic bis(dipyrrinato)zinc(II) complexes. The degeneration of the frontier orbitals (highest π and lowest π* orbitals of the dipyrrinato ligand) in the heteroleptic complex was ignored. Figure 15b shows one of the possible energy diagrams for the heteroleptic complex. In this case, the two CS states would be less energetically favorable compared with the emissive ^1^π-π* state, so the fluorescence would be enhanced.

The heteroleptic complex could also have another frontier orbital configuration, as shown in Figure 15c. In this case, one of the two CS states would be stabilized over the emissive ^1^π-π* state, resulting in the quenching of the fluorescence. This phenomenon would be unfavorable, but the observation of the enhancement and depression of the fluorescence in heteroleptic complexes would support our hypothesis that thermally accessible CS states are responsible for the low fluorescence quantum yield in the zinc(II) complex.

**Figure 16:**
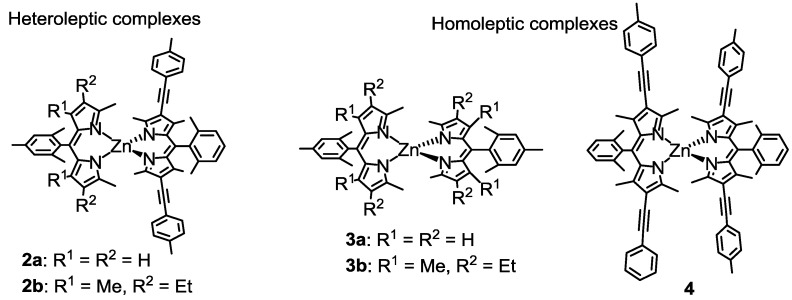
Homoleptic bis(dipyrrinato)zinc(II) complexes **2a** and **2b**, and corresponding homoleptic complexes **3a**, **3b**, and **4**.

In this work, we synthesized two heteroleptic bis(dipyrrinato)zinc(II) complexes, **2a** and **2b** (Figure 16) [95]. Their synthesis, photochemical properties, and frontier orbital ordering were discussed, and compared with the corresponding homoleptic complexes **3a**, **3b**, and **4** (Figure 16).

### 2.3. Synthesis, Structure, and Thermal Stability

**Scheme 1:**
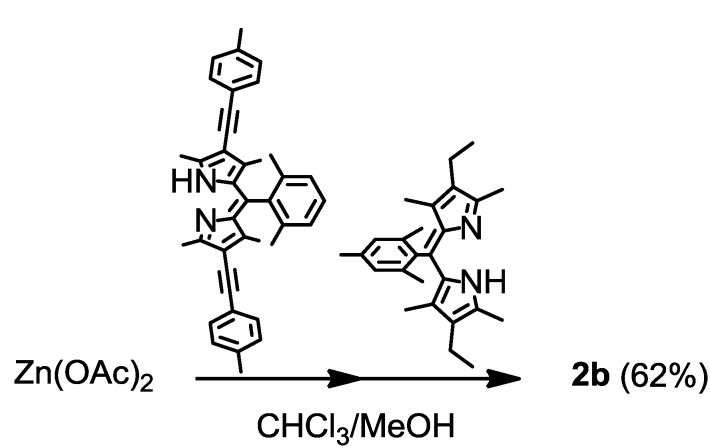
Synthesis of **2b**.

The heteroleptic complexes were synthesized using the stepwise coordination method. Chloroform solutions of the two dipyrrin ligands were added stepwise to zinc(II) acetate in methanol, at room temperature (Scheme 1). The isolation yield exceeded the statistical value of 50%.

**Figure 17:**
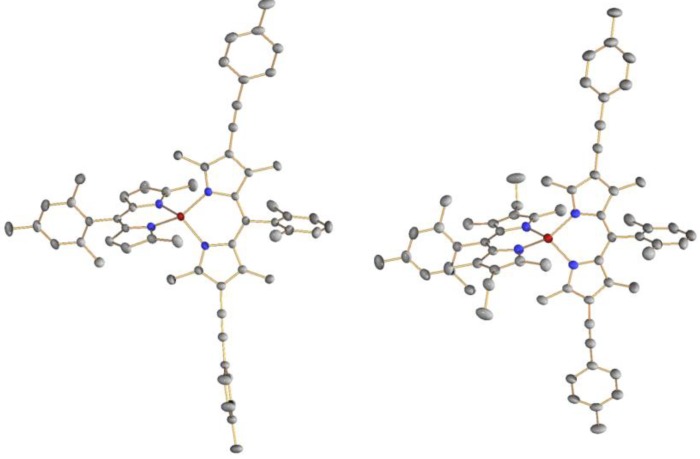
ORTEP drawings of **2a** (left) and **2b** (right), with a thermal ellipsoid set at the 50% probability level. Hydrogen atoms are omitted for clarity.

Single crystal X-ray crystallographic analysis was conducted to reveal the precise structures of the bis(dipyrrinato)zinc(II) complexes. ORTEP drawings of **2a** and **2b** are shown in Figure 17. The two dipyrrin ligands were almost perpendicular to each other (dihedral angles: 88° and 87°), indicating that the zinc center was tetrahedral, as observed in homoleptic complexes [96].

We investigated the thermal stability of the heteroleptic complexes against disporporionation into the corresponding homoleptic complexes, as follows. A degassed dichloromethane-*d*_1_ solution of **2a** was sealed in an NMR tube, and heated at 100 °C for 1 day, in the dark. This treatment did not produce any change in the ^1^H NMR spectrum, which indicated the good stability of the heteroleptic form.

### 2.4. Optical Properties

**Figure 18:**
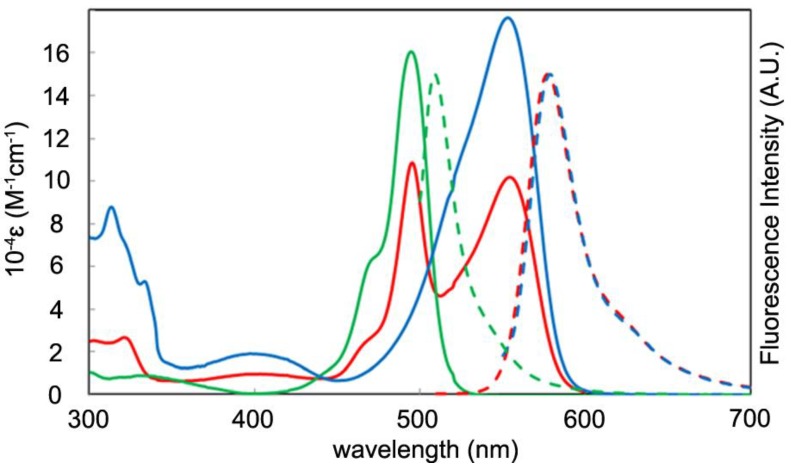
Absorption and emission spectra for **2a**, **3a**, and **4** in toluene.

Figure 18 shows the absorption and fluorescence spectra for **2a**, **3a**, and **4**; the associated numerical data, including the data for **2b** and **3b**, are presented in Table 1. Homoleptic complexes **3a** and **4** showed a single strong absorption band in the visible region, which was assigned to the ^1^π-π* transition of the dipyrrinato ligands. The introduction of arylethynyl groups in **4** resulted in a redshift of the ^1^π-π* band, compared with **3a**. The absorption spectrum of heteroleptic **2a** was the average of those of **3a** and **4**. This fact indicated that there were no appreciable interactions in the ground state in the heteroleptic complex.

**Figure 19:**
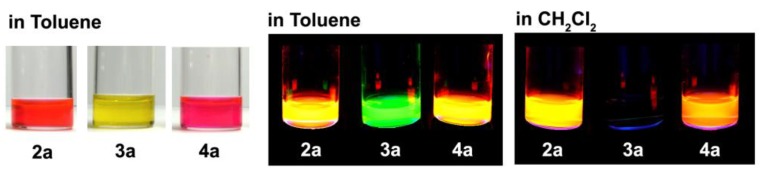
Photographs of solutions of **2a**, **3a** and **4** under daylight (left), and UV irradiation (middle and right).

In contrast, several distinctive phenomena were observed in the emission spectra. Homoleptic **3a** and **4** showed one emission maximum corresponding to the ^1^π-π* fluorescence, but heteroleptic **2a** showed only one emission band at 578 nm, similar to **4**, even upon excitation with the ^1^π-π* band of the left-hand dipyrrinato ligand. This suggested that **2a** underwent a quantitative intraligand energy transfer from the left-hand dipyrrinato ligand to the right-hand one. The most important feature of heteroleptic **2a** was its higher fluorescence quantum yield compared with homoleptic **3a** and **4** (Table 1 and Figure 19). The superiority of heteroleptic **2a** as a fluorophore over homoleptic **3a** and **4** was more significant in the more polar solvent, dichloromethane (Table 1 and Figure 19). We note that the fluorescence quantum yield of **2a** exceeded those of the corresponding BODIPYs.

We did find, however, that a heteroleptic structure was not a prerequisite for high fluorescence efficiency. **2b** showed much smaller fluorescence quantum yields than the corresponding homoleptic complexes and the heteroleptic **2a** (Table 1 and Figure 19).

**Table 1:** Photochemical properties of **2a**, **2b**, **3a**, **3b**, and **4**.

	**10^−4^*ε* (M^−1^ cm^−1^)**	***λ*_abs_ (nm)**	***λ*_em_ (nm)**	***φ*_F_ (in Toluene)**	***φ*_F_ (in CH_2_Cl_2_)**
2a	11, 10	495, 553	578	0.76, 0.75	0.53, 0.51
2b	11, 10	508, 556	579	0.07, 0.08	0.01, 0.03
3a	16	495	509	0.28	0.00
3b	14	508	532	0.20	0.05
4	18	553	579	0.72	0.31

### 2.5. DFT Calculations

**Figure 20:**
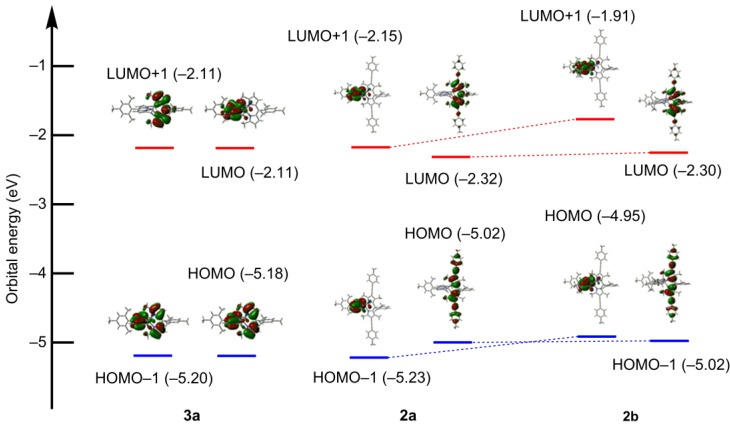
Frontier orbitals of **3a** (left), **2a** (middle), and **2b** (right), calculated using the B3LYP/6-31G(d) level of theory, considering CH_2_Cl_2_ solvation using the PCM method.

To rationally explain the photoproperties of the heteroleptic complexes, we conducted DFT calculations (Figure 20). Homoleptic complex **3a** had degenerated HOMOs and LUMOs corresponding to the π and π* orbitals of the two identical dipyrrinato ligands (Figure 20a). The degeneration of the frontier orbitals was suppressed in **2a**; the HOMO and LUMO were localized on the right-hand ligand, and the HOMO-1 and LUMO+1 were localized on the left-hand ligand. This was a good electronic structure for the improvement of the fluorescence quantum yield, assuming the charge-separation hypothesis (Figure 15b). However, compared with **2a**, the order of HOMO and HOMO-1 was inverted in the heteroleptic **2b**. In the charge-separation hypothesis, such an electronic structure would stabilize the charge-separated state, resulting in the reduction of the fluorescence quantum yield (Figure 15c).

### 2.6. Conclusions

This chapter presented details of the synthesis, structure, and optical properties of heteroleptic bis(dipyrrinato)zinc(II) complexes. A great improvement in the fluorescence quantum yield was achieved, which was well explained by the charge-separation hypothesis. These findings will increase the range of applications for bis(dipyrrinato)zinc(II) complexes, especially in the photochemical field.

## 3. New Discoveries for Pechmann Dyes: An Alternative Synthesis, a Missing Structural Isomer, and Applications for Organic Electronics

### 3.1. Introduction

**Scheme 2:**
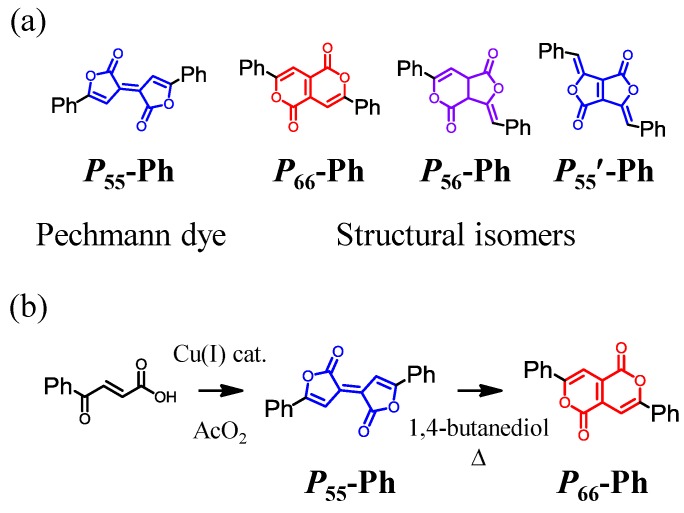
(**a**) Pechmann dye (***P*_55_-Ph**) and its structural isomers (***P*_66_-Ph**, ***P*_56_-Ph**, and ***P*_55’_-Ph**, and (**b**) representative synthetic route for ***P*_55_-Ph** and ***P*_66_-Ph**.

Pechmann Dye, *E*-5,5'-diphenylbifuranylidenedione (***P*_55_-Ph**, Scheme 2a), was first reported by von Pechmann and coworkers in 1882 [97]. This series of molecules possesses good photochemical properties, including an intense absorption at approximately 550 nm, which is ascribed to a ^1^π-π* transition, and intense ^1^π-π* fluorescence [98]. Rahmani and coworkers revealed its structure via single crystal X-ray structural analysis [99]; a noteworthy feature is its planar structure. Good thermal stability in the solid state and electron acceptor abilities are other distinctive virtues of Pechmann dye. One of the representative synthetic routes for Pechmann dye is shown in Scheme 2(b). The dimerization of β-aroylacrylic acid in acetic anhydride, in the presence of cuprous chloride as a catalyst, produced Pechmann dye in good yields [100]. Its structural isomer, 3,7-diphenylpyrano[4,3-c]-pyran-1,5-dione (***P*_66_-Ph**, Scheme 2a) was produced by thermal isomerization under basic conditions in the Pechmann dye [101]. ***P*_66_-Ph** is also a good dye molecule, showing a strong absorption at approximately 430 nm [102]. There are two more possible isomeric forms of Pechmann dye, ***P*_56_-Ph** and ***P*_55_’-Ph** [Scheme 2(a)]. However, the synthesis of these isomers has not yet been accomplished.

Recently, **D-A-D** and **A-D-A** (**D**: donor, **A**: acceptor) quadrupolar types of molecules have attracted the attention of chemists. These types of molecules feature intense one-photon absorptions in the visible and NIR regions, which are due to intramolecular charge-transfer (ICT) transitions, high fluorescent quantum yields, and large two-photon absorption cross sections. These features make **D-A-D** and **A-D-A** quadrupolar molecules promising materials for applications such as bioimaging [103,104], photodynamic therapy (PDT) [105], optical power limiting [106,107], organic light emitting diodes (OLEDs) [108,109,110], organic photovoltaic devices (OPVs) [111,112], and organic field effect transistors (OFETs) [113,114]. The electron acceptor abilities of Pechmann dye and its structural isomers make them excellent components for the **D-A-D** type of quadrupolar molecules; despite this, such systems have not yet been investigated.

The present study aimed to solve the remaining problems associated with Pechmann dye. We developed a new synthetic procedure for Pechmann dye, which enabled us to synthesize one of the missing structural isomers, ***P*_56_-Ar**. By employing triarylamine as the aryl group, we fabricated **D-A-D** quadrupolar molecules based on Pechmann dyes and demonstrated their good dye abilities, as well as their potential for applications in organic electronics [115].

### 3.2. Synthesis, Identification, and Reaction Mechanism

**Scheme 3:**
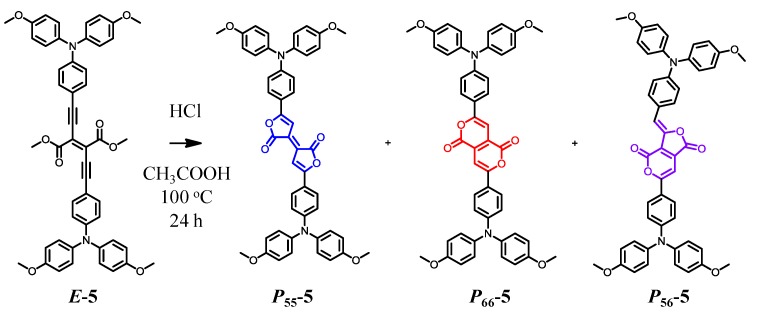
(**a**) Add a descriptive label of the figure here. (**b**) Add a descriptive label of the figure here. (**c**) Add a descriptive label of the figure here.

**Figure 21:**
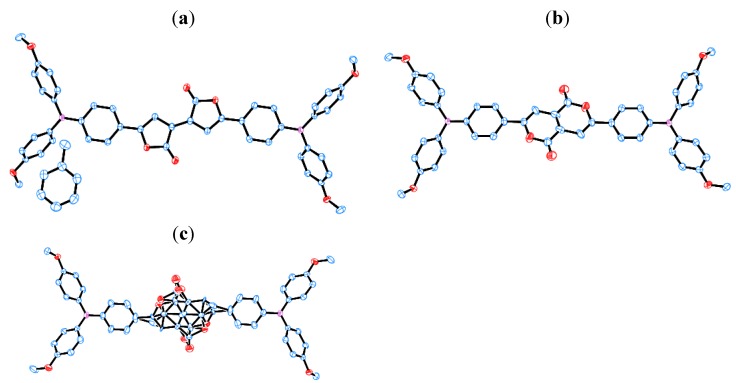
ORTEP drawings of (**a**) ***P*_55_-5·toluene**, (**b**) ***P*_66_-5**, and (**c**) ***P*_56_*-*5** with thermal ellipsoids set at the 50% probability level. ***P*_56_*-*5** comprises two disordered structures. Hydrogen atoms are omitted for clarity.

Previously, we investigated the dimethyl diethynylfumarate framework (Scheme 3) [116,117,118]. For example, with ***E*-5** forming the framework and the presence of triarylamine, interesting photochemical activities were produced, including strong absorption in the visible region, reversible visible-light *E*-*Z* photoisomerization accompanied by the switching of the fluorescence, and electronic communication in the one-electron oxidized MV state. In the present work, we regarded the dimethyl diethynylfumarate framework as the open form of Pechmann dye. We expected that with appropriate ring closing reactions between the ethynylene and carboxyl group [119,120], the dimethyl diethynylfumarate framework could be converted into the Pechmann dye frameworks, including the missing structural isomers. Scheme 3 shows a reaction condition for the conversion of ***E*-5** into the Pechmann dye derivatives ***P*_55_-5**, ***P*_66_-6**, and ***P*_56_*-*5**. The treatment of ***E*-5** with hydrochloric acid in acetic acid at 100°C produced three compounds with different colors (blue, red, and purple). Single-crystal X-ray structure analysis revealed their structures (Figure 21). The three compounds were identified as blue ***P*_55_-5** (yield: 11%), red ***P*_66_-6** (43%), and purple ***P*_56_*-*5** (1%). It is noteworthy that ***P*_56_*-*5** comprised one of the missing structural isomers of Pechmann dye.

**Scheme 4:**
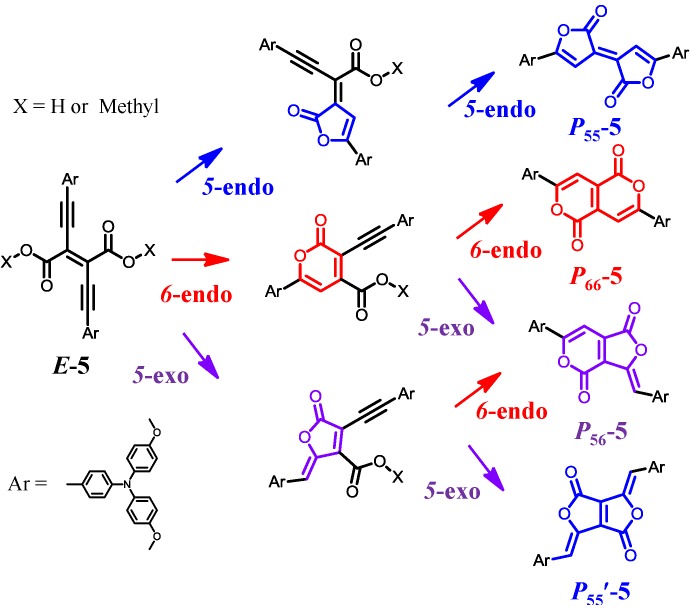
Plausible reaction mechanism for the generation of ***P*_55_-5**, ***P*_66_-5**, and ***P*_56_*-*5** from ***E*-5**.

Scheme 4 shows a plausible reaction mechanism for ***P*_55_-5**, ***P*_66_-5**, and ***P*_56_*-*5**. They were generated via intramolecular double lactionization. The lactonication could be classified into three types, ***5*-endo**, ***6*-endo**, and ***5*-exo**. Judging from the fact that ***P*_55_'-5** was not obtained at all, and the yield of ***P*_56_*-*5** was the lowest among the three compounds, the ***5*-exo** cyclization was least favorable among the three lactonization modes.

### 3.3. Photochemical Properties

**Figure 22:**
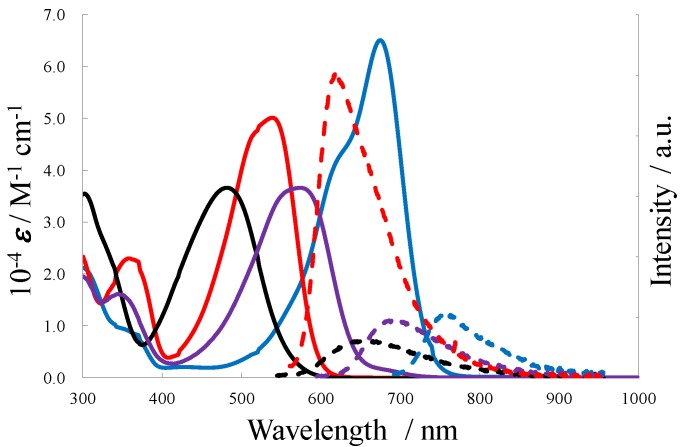
UV-vis-NIR (solid) and fluorescence (dashed) spectra for ***E*-5** (black), ***P*_55_-5** (blue), ***P*_56_-5** (purple), and ***P*_66_-5** (red) in toluene.

Figure 22 shows the absorption and fluorescence spectra for ***E*-5**, ***P*_55_-5**, ***P*_66_-5**, and ***P*_56_-5** in toluene, and Table 2 details their numerical data. DFT calculations were used to assign the most intense absorption bands in the visible and NIR regions to intramolecular charge transfer (ICT) transitions from the donor (chiefly distributed on the n orbital of the triarylamine moiety) to the acceptor (the π* orbital of the dimethyl diethynylfumarate or dilaction framework). The lactonized compounds showed red-shifts of the ICT bands relative to the parental compound, ***E*-5**: ***P*_66_-5** (0.53 eV), ***P*_56_-5** (0.77 eV), and ***P*_55_-5** (1.03 eV). This was ascribed to the lowering of the π* orbital level upon the double lactonization. The absorption for ***P*_55_-5** and ***P*_66_-5** occurred at longer wavelengths than that for ***P*_55_-Ph** and ***P*_66_-Ph**, because of the existence of the triarylamine moiety, and the expression of the **D-A** interaction. The fluorescence quantum yields (***Φ***_F_) were measured to be 0.27 for ***P*_55_-5**, 0.20 for ***P*_56_-5**, 0.82 for ***P*_66_-5**, and 0.15 for ***E*-5**. The lactonized compounds showed brighter fluorescence than the parental ***E*-5**.

**Table 2:** Photochemical data for ***E*-5**, ***P*_55_-5**, ***P*_56_-5**, and ***P*_66_-5** in toluene.

	10^−4^*ε* (M^−1^ cm^−1^)	*λ*_abs_ (nm)	*λ*_em_ (nm)	*φ*_F_
*P*_55_-5	65	674	753	0.27
*P*_56_-5	37	574	692	0.20
*P*_66_-5	50	538	617	0.82
*E*-5	36	482	660	0.15

### 3.4. Application to Organic Electronics

**Figure 23:**
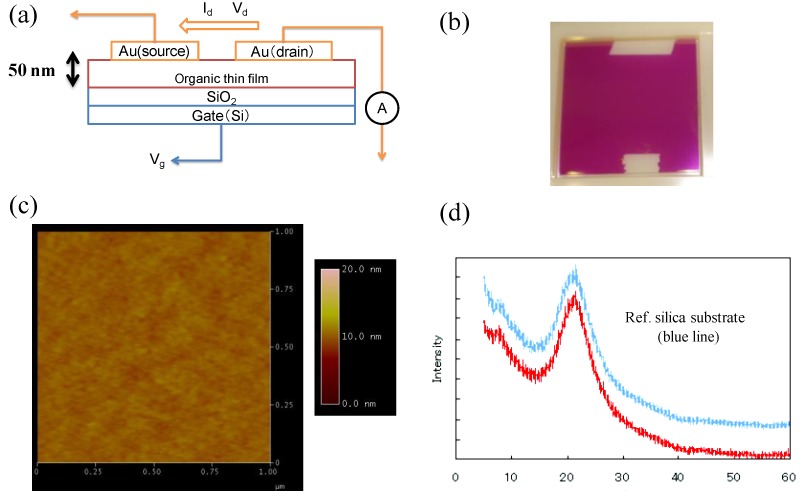
(**a**) Schematic illustration of a top-contact OFET device. (**b**) Photograph of the ***P*_66_-5** thin film. (**c**) AFM image of a ***P*_66_-5** thin film on a quartz substrate. (**d**) XRD patterns for the ***P*_66_-5** thin film on a silicon substrate (red) and on a bare substrate (blue).

To demonstrate the utility of Pechmann dyes, we investigated a preliminary OFET device based on ***P*_66_-5** (Figure 23a). ***P*_66_-5** was subjected to vacuum deposition at 8.2 × 10^−5^ Pa, 468 K, and a rate of 1.3 Ås^−1^ so that a flat and smooth thin film of ***P*_66_-5** was fabricated on silicon substrates modified with hexamethyldisilazane (HMDS) (Figure 23b). The thickness of the deposited film, *ca.* 50 nm, was confirmed by an AFM analysis, where the film was deposited on a quartz substrate under the same condition as described above (Figure 23c). On the other hand, XRD analysis for the thin film on the silicon substrate disclosed its amorphous nature (Figure 23d). Gold electrodes as source and drain were attached on the thin film of ***P*_66_-5** to complete the top-contact OFET configuration, where the channel width and channel length was 3 cm and 50 μm, respectively (Figure 23a). The device characteristics were investigated under an ambient condition. The thin film of ***P*_66_-5** functioned as a p-type semiconductor, with a maximum hole mobility of 5.6 × 10^–5^ cm^2^ V^–1^ s^–1^. This is the first report of the application of Pechmann dyes for organic electronics. The carrier mobility could be enhanced by improving the low crystallinity of the ***P*_66_-5** thin film. 

## 4. Conclusions

We developed an alternative synthetic route for Pechmann dyes. The dimethyl diethynylfumarate framework was converted into Pechmann dyes, including one of the missing structural isomers (***P*_56_-Ph**), via intramolecular double lactonization. We synthesized **D-A-D**-type quadrupolar molecules ***P*_55_-5**, ***P*_66_-5**, and ***P*_56_-5** using triarylamine as the donor and Pechmann dyes as the acceptor. The quadrupolar molecules showed intense absorption and fluorescence in the visible and NIR regions, which was ascribed to ICT transitions from the donor to the acceptor. We fabricated an OFET device using ***P*_66_-5** as an active layer, revealing the potential of Pechmann dyes for organic electronics.
